# Enhancing cardiovascular risk prediction through AI-enabled calcium-omics

**DOI:** 10.1038/s41598-024-60584-8

**Published:** 2024-05-15

**Authors:** Ammar Hoori, Sadeer Al-Kindi, Tao Hu, Yingnan Song, Hao Wu, Juhwan Lee, Nour Tashtish, Pingfu Fu, Robert Gilkeson, Sanjay Rajagopalan, David L. Wilson

**Affiliations:** 1 Department of Biomedical Engineering, Case Western Reserve University, Cleveland, OH 44106 USA; 2 Harrington Heart and Vascular Institute, University Hospitals Cleveland Medical Center, Cleveland, OH 44106 USA; 3 School of Medicine, Case Western Reserve University, Cleveland, OH 44106 USA; 4 Department of Population and Quantitative Health Sciences, Case Western Reserve University, Cleveland, OH 44106 USA; 5 Department of Radiology, University Hospitals Cleveland Medical Center, Cleveland, OH 44106 USA; 6 Department of Radiology, Case Western Reserve University, Cleveland, OH 44106 USA

**Keywords:** Calcification, Cardiovascular biology, Atherosclerosis

## Abstract

Whole-heart coronary calcium Agatston score is a well-established predictor of major adverse cardiovascular events (MACE), but it does not account for individual calcification features related to the pathophysiology of the disease (e.g., multiple-vessel disease, spread of the disease along the vessel, stable calcifications, numbers of lesions, and density). We used novel, hand-crafted calcification features (calcium-omics); Cox time-to-event modeling; elastic net; and up and down synthetic sampling methods for imbalanced data, to assess MACE risk. We used 2457 CT calcium score (CTCS) images enriched for MACE events from our large no-cost CLARIFY program (ClinicalTrials.gov Identifier: NCT04075162). Among calcium-omics features, numbers of calcifications, LAD mass, and diffusivity (a measure of spatial distribution) were especially important determinants of increased risk, with dense calcification (> 1000HU, stable calcifications) associated with reduced risk Our calcium-omics model with (training/testing, 80/20) gave C-index (80.5%/71.6%) and 2-year AUC (82.4%/74.8%). Although the C-index is notoriously impervious to model improvements, calcium-omics compared favorably to Agatston and gave a significant difference (*P* < 0.001). The calcium-omics model identified 73.5% of MACE cases in the high-risk group, a 13.2% improvement as compared to Agatston, suggesting that calcium-omics could be used to better identity candidates for intensive follow-up and therapies. The categorical net-reclassification index was NRI = 0.153. Our findings from this exploratory study suggest the utility of calcium-omics in improved risk prediction. These promising results will pave the way for more extensive, multi-institutional studies of calcium-omics.

## Introduction

There is a great need for precision risk tools to guide personalized prevention strategies for heart health. While cardiovascular risk can be estimated using many widely available cardiovascular risks scores from clinical factors, most scores suffer from poor discrimination^1^. The CT calcium score (CTCS) imaging exam can provide direct evidence of coronary atherosclerosis when calcifications are present in the coronary arteries and is acknowledged by several guidelines as a preferred risk assessment tool^2,3^. The presence of coronary artery calcium (CAC) is by far the best predictor of future major adverse cardiovascular events (MACE) outperforming every other risk factor and composite clinical risk scoring approaches. The addition of CAC score to traditional risk factors has been shown to consistently improve discrimination and reclassification^4^. Despite their acknowledged superiority over current risk assessment approaches, current approaches for CAC-based risk prediction are overly simplistic and suffer from a number of limitations. The Agatston method simply uses a non-linear weighted sum of the areas of coronary artery calcium (CAC) with densities above 130 HU. A calcium mass score is known to be more reproducible^5^. Importantly, current CAC scoring approach ignores a plethora of other CAC features that may be pathophysiologically important, including density, distribution, geometry, and others. Some alternatives have appeared in the literature (e.g., spatial distribution, diffuse CAC, and high-density calcified plaque^6–8^) but never in combined fashion. Other pathophysiologic observations on calcifications have suggested a number of aspects that could be important but are currently not incorporated in the analysis of CTCS images.

The use of CTCS imaging has been re-invigorated with AHA guideline recommendations for the test. Many sites offer low-cost exams, and our institution offers a no-cost CT calcium score exam. As a result, large data sets are being accrued, buoying our interest in creating a more intensive AI analysis approach than has been done to date.

In this work, we evaluate a novel machine approach that includes numerous hand-crafted features aimed at capturing pathophysiology of atherosclerotic calcifications (calcium-omics) and evaluate their collective ability to predict MACE in time-to-event models. Our calcium-omics includes for the first-time combinations of shape, mass, density, volume, number of calcifications, diffusivity, and others which together could potentially better capture a patient’s risk of a MACE event. We use Cox time-to-event modeling, elastic net, up and down synthetic sampling methods for imbalanced data, and determine our ability to separate low and high-risk groups. In addition to determining risk from combined calcium-omics, we use Cox models on individual features and subsets of features in order to determine explainable high-risk characteristics in the images.

## Methods

### Study data

Non-contrast CTCS images were acquired from a variety of CT scanners using 120-kVp, nominally 30-mAs, with an average 0.5 × 0.5-mm in-plane voxel spacing and 2.5-mm slice thickness. A typical CTCS volume consists of 40 slices of 512 × 512 voxels, giving 10.5 million voxels per volume. We used CTCS images from 2457 patients (single CTCS volume per patient) enriched for MACE (13.8%), with characteristics in Table 1. MACE was defined as first event of myocardial infarction, stroke, coronary revascularization, or all-cause mortality. Cardiovascular outcomes were obtained from the UH CLARIFY study with a maximum of 6 years of follow-up (mean follow-up is 1.9 years). The included population had not experienced MACE, including revascularization, before the CTCS exam. The CTCS images utilized in this study were accompanied by manual segmentations conducted by experts from our institution as part of the clinical routine. The experts identified coronary calcifications and excluded other calcifications (e.g., aortic valve, aortic, or pulmonary artery calcifications). Experts excluded any stent, pacemaker, or other man-made objects. Patient’s MACE-free time is reported as the duration from the start time (time of CTCS exam) until the patient either had MACE or was censored (left the study or survived to the end of observation without MACE). This study on de-identified data was approved by the Institutional Review Board (IRB) of the University Hospitals Cleveland Medical Center. All scans in this study were obtained as part of clinical care and informed consent was obtained from all subjects and/or their legal guardian(s). Methods were carried out in accordance with relevant guidelines and regulations.

**Table 1:** Characteristics of our randomly chosen cohort of 2457 enriched with regards to MACE events.

Characteristic	Full cohort, N = 2457	No-MACE, N = 2118	MACE, N = 339	*P* value
**Demographics**				
Age, y *	60.7 ± 9.6 (19, 90)	60 ± 9.5 (19,90)	65.6 ± 8.5 (41,87)	< 0.0001***
Women	1185 (48.2%)	1044 (49.3%)	141 (41.6%)	0.008*
BMI, kg/m^2^ (missing 320)	30 ± 6.4, N = 2327	29.88 ± 6.4, N = 1828	30.65 ± 6.2, N = 309	0.045*
**Risk factors and medications**				
MACE	339 (13.8%)	–	–	–
Time within study, d, y*	699 (3, 2192) days	726 (31, 2192)	530.5 (3,2170)	< 0.0001***
	1.9 (0, 6) years	2 (0, 6)	1.5 (0, 5.9)	
10-year risk, pooled cohort equations (PCE), %	13.04% ± 11.5, N = 1468	11.9% ± 10.8, N = 1275	20.64% ± 13.5, N = 193	< 0.0001***
Diabetes	387 (15.8%)	287 (13.6%)	98 (28.9%)	< 0.0001***
Smoking	757 (30.8%)	569 (26.9%)	188 (55.5%)	< 0.0001***
**Baseline measurements**				
HDL-cholesterol, mg/dL	53.95 ± 15.9, N = 1565	54.25 ± 16, N = 1361	51.92 ± 15.2, 204	0.043*
LDL-cholesterol, mg/dL	117.4 ± 38, N = 1591	118.56 ± 37.5, N = 1385	109.73 ± 40.4, N = 206	0.003**
Zero Ag score	957 (38.9%)	942 (44.5%)	15 (4.4%)	< 0.0001***
Agatston score*	220 ± 364.4(0, 1992)	190.9 ± 337.2 (0, 1992)	402.2 ± 462.6 (0, 1969)	< 0.0001***
Mass score*	33.9 ± 56.4 (0, 342)	29.4 ± 51.7 (0, 327.8)	62.4 ± 73.6 (0, 342)	< 0.0001***
Volume score*	186.3 ± 299.2 (0, 1819.7)	161.3 ± 275.7 (0, 1819.7)	342 ± 382.1 (0, 1776.8)	< 0.0001***
Num lesions*	5 ± 6.9 (0,65)	4.5 ± 6.4 (0, 46)	9 ± 8.1 (0, 65)	< 0.0001***

Characteristics are given for the full cohort and the MACE and no-MACE groups. The cohort has great variability along clinical features (female and gender) high percent of zero Agatston score (38.9%). The image-driven score features such as Agatston, mass, and volume score, in addition to the total number of lesions, show good statistical distributions. MACE vs. no-MACE sub-cohorts shows great stratification along all features.

Numbers reported as mean ± standard deviation (min, max) values.

*P*-values are star-coded based on the significance levels as follows: (< 0.0005 as ***, < 0.005 as **, < 0.05 as *).

### Image analysis and risk prediction methods

#### Data preparation

**Figure 1:**
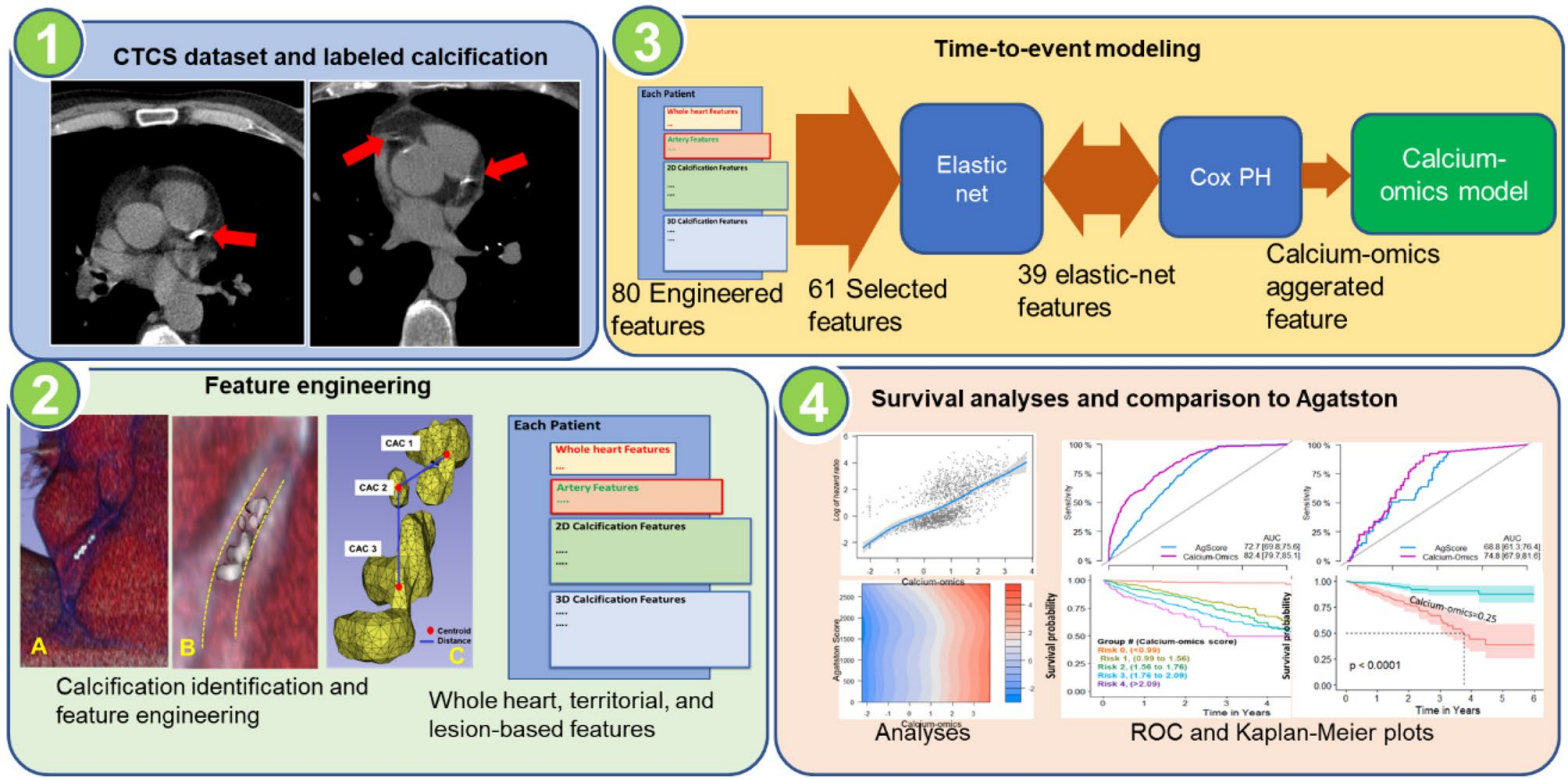
MACE prediction using calcium-omics features pipeline. In (1), CAC lesions in CTCS images were analyzed and labeled using semi-automated commercial software. In (2), calcium-omics features were engineered and categorized based on whole heart, territorial, and lesion features. In (3) MACE risk prediction model was designed using elastic-net and Cox model. In (4), results and statistical analyses were performed to assess the importance of our novel calcium-omics model compared to variety of univariate and multivariable Cox models.

Previously, patient images were analyzed using semi-automated commercial software. The criteria for calcification detection were according to prior standards endorsed by guidelines which specified three connected voxels with HU $\ge$ 130^9^. Analysts went through each volume, slice-by-slice, and assigned each coronary calcification to a territory. For each heart, the software created a mask volume, identifying the calcifications in each territory with a different color and computed whole heart as well as territorial Agatston score. We excluded cases that had (1) poor image quality and (2) showed > 10% Agatston score difference between commercial and automated in-house deep learning software. As a preprocessing step, the color-coded masks were deciphered to obtain the proper territory, creating a clean mask volume. This step required special processing to ignore extra text labels embedded in the image. The pipeline of our proposed model is shown in Fig. 1.

#### Calcium-omics feature engineering

**Figure 2:**
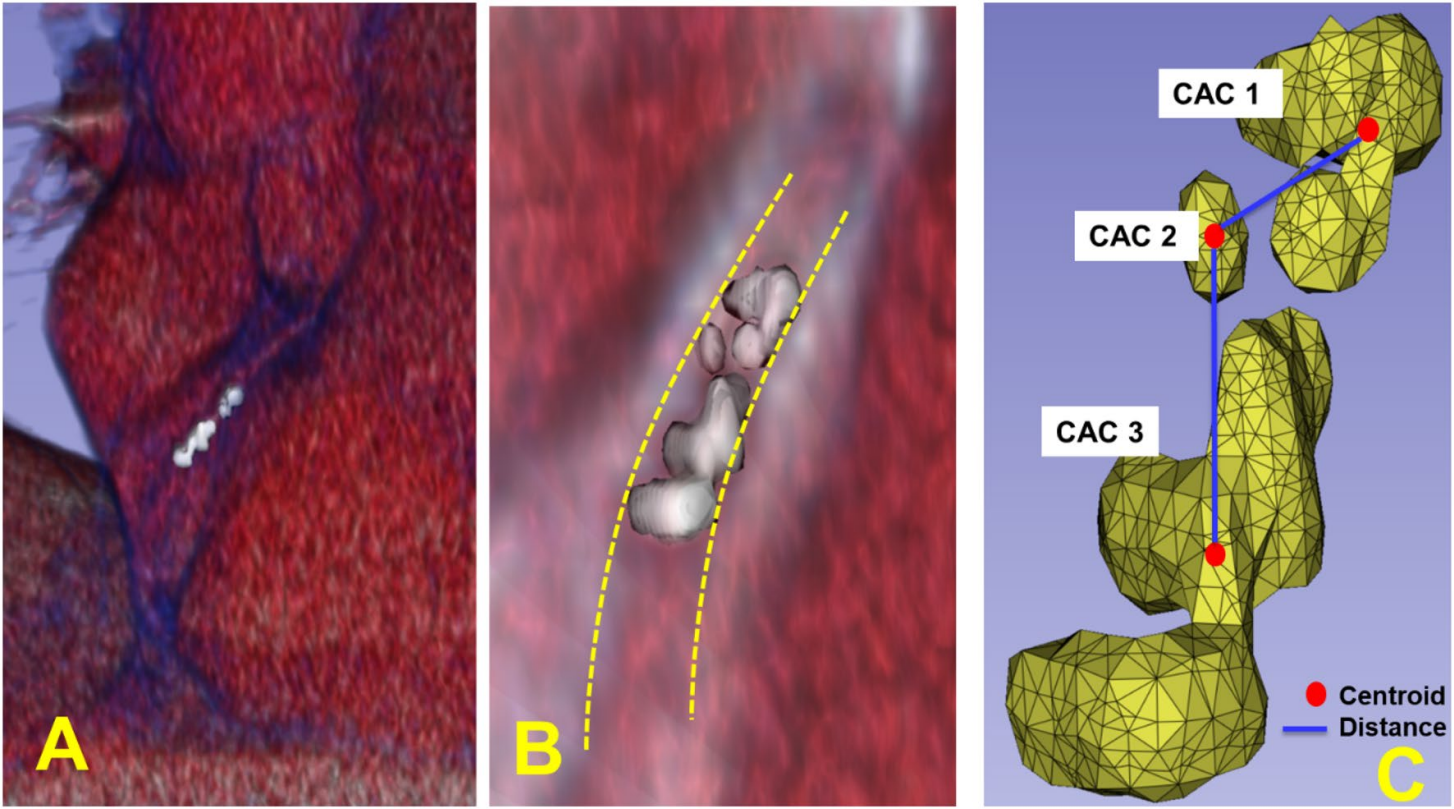
Individual calcifications and engineered calcium-omics features. Three consecutive calcifications in the LCX artery territory are shown in (**A**) and magnified in (**B**), where dashed lines annotate the vessel wall. Calcification masks are rendered in (**C**). Some features are aggregated along each artery, such as Agatston, mass, and volume scores, which give this LCX artery 84.4, 13.3, and 73.2, respectively. Calcification centroids are used to calculate the Euclidean distances between calcifications. The sub-voxel centroid (x, y, z) locations are used to calculate the calcified arterial distance to sum DistFirst2LastLesionPerArtery from a centroid to a centroid in consecutive sequential order. An example of a new feature is “DistFirst2LastLesionPerArtery_LCX” which represents the total Euclidean distance along lesions within LCX.

Using the mask volume as a guide, we created software to compute various calcium-omics features for each individual calcification, artery territory, and whole heart. For each individual calcification, we collected elemental features including mass, volume, territory, HU values, first moment, second moment, shape, distance to a subsequent lesion, distance to the top of the CT volume, artery diffusivity, among others. (Artery diffusivity was the ratio of number of calcified lesions to the Euclidian distance from first to last lesion within an artery) and represents the distribution of lesions within artery. For a territory with no calcifications or a single calcification, we set diffusivity to 0 and 1, respectively. In addition, additional statistical features such as mean, standard deviation, skewness, kurtosis, and small histogram were obtained per territory and for the entire heart. In total, we collected 80 calcium-omics features. Agatston, mass, and volume were obtained at the level of individual calcification, coronary territory, and whole heart levels. As demonstrated in Fig. 2, different features were aggerated within three levels (lesion calcification, artery, and whole heart). Details of calcium-omics features, and time-to-event modeling are described in the supplemental file.

#### MACE risk prediction and performance evaluation

We randomly divided data into training/held-out-testing subsets with 80:20 ratio for all our experiments, maintaining a similar MACE-event ratio for training and testing sets. We used the natural logarithm function to condense features with broad-range values (e.g., Agatston and mass scores). Starting with 80 calcification features, we eliminated 19 irrelevant or highly autocorrelated features by univariate Cox modeling, leaving 61 features (Table S1).

To determine high risk features and enhance explainability of results, we investigated selected univariate and multivariable Cox models. We evaluated the impact of mass scores using multivariable Cox models and investigated the impact of adding features such as the number of lesions, max HU, distance-based along territories, and CAC distribution along territories (diffusivity) to the mass score model. As a machine learning technique, Cox modeling provides interpretable results that can explain the effect of those imaging features which would be unavailable in deep learning. To enhance comparisons, all Cox models in our study were trained and tested on the same data.

We selected the most informative and non-correlated features using elastic-net as implemented in R package $glmnet$. Elastic-net was performed on the training subset using tenfold cross-validation, with $\alpha =0.05$, and $\lambda =0.074$, where these parameters were determined in preliminary evaluations. Out of the 61 engineered features, elastic net selected 39 features with non-zero coefficients ($\beta$). Features include whole heart features (e.g., mass score, volume score, and number of lesions), territorial features (e.g., mass score in LAD, number of lesions in RCA, and distance from top to last lesion in LCX), and calcification features (e.g., mass histogram bin, the maximum first momentum value of individual calcification, and the third skewness value of individual calcification), as shown in table S1. These features were aggregated into a single “calcium-omics” feature by summing the products of these features by their corresponding coefficients. We used R 4.2.1^10^, the Cox model package $coxph( )$, and elastic-net package $glmnet( )$.

To evaluate the performance of those models, we utilized multiple time-to-event analyses. Standard metrics included C-index, AUC at fixed time points, and log-rank score. As C-index is notoriously incentive to model improvements^11,12^, we used other metrics to evaluate performance. Hazard ratios with confidence intervals are presented so as to isolate the impact of a single feature. In addition, we stratified risk groups and created Kaplan–Meier (KM) plots. We also computed categorical net reclassification improvement. In some instances, we compared groups using student’s t-test, with significant differences identified when *p* < 0.05.

## Results

### Data analysis and model subsets to identify high risk features

**Figure 3:**
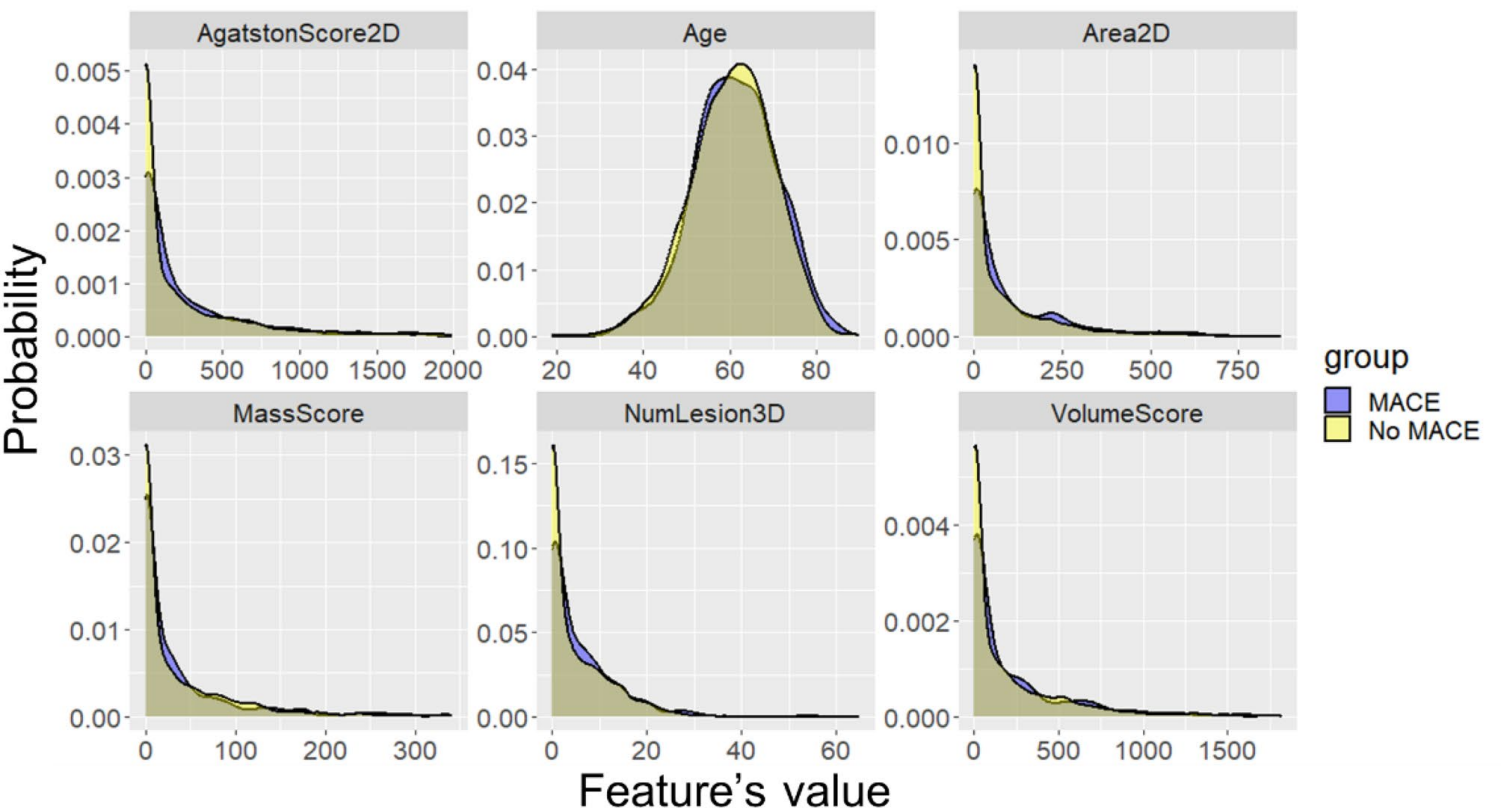
Normalized histograms of feature values for the MACE and no-MACE groups. For the 80 features we analyzed, we found that no single feature, including Agatston and mass scores, gave strong visual evidence of differences between groups. However, because of the large number of samples, t-tests gave *p* < 0.0001, allowing us to reject the null hypothesis of no difference in the means. Of course, these histograms do not consider censoring as done with time-to-event modeling. The x-axis represents the values of each feature, while the y-axis is the probability of histogram bins as obtained by normalizing the histogram.

Histograms of selected calcium-omics features are shown in Fig. 3. Distributions for MACE and no-MACE have considerable overlap, eliminating the possibility of creating clear-cut decision rules for MACE based on single feature, with the exception of zero total calcium score. The lack of clear discriminating thresholds suggests the need for an AI approach using multiple features at once.

We investigated multiple univariate and multivariable Cox models to understand and explain the role of particular features on MACE prediction (Table 2). Comparing Agatston (line 1) and mass score (line 2), we determined that mass score had a slightly higher C-index and AUC at 2 years. As the mass score is generally considered more reproducible than Agatston^5,13^, we used it in subsequent evaluations. When we examined territorial mass scores (line 3), we found improved discrimination (C-index and AUC) compared to a whole heart mass score particularly for the LAD, which has the lowest *p*-value and the highest HR, indicating that an equivalent mass in the LAD was more predictive of MACE than that in another territory. For a given mass score, increasing the number of lesions had a significant effect (*p* < 0.004) (line 4). For every unit increase of (ln(1 + NumLesions)), the risk of having MACE increased by 1.48-fold. Hence, compared to one lesion, the risk was increased by 227% for 40 lesions, a number sometimes observed. Adding a logical feature to indicate two or more territories with calcification did not improve mass score model (*p* = 0.12) (line 5 versus line 2). In contrast, for a given mass score, HU > 1000 was protective (line 6). The distance from the “top” to “bottom” calcification per territory (line 7) improved performance with regard to log-rank score, C-index, and AUC as compared to other models (lines 1–6), even though no single territory produced a significant effect (*p* < 0.05). The number of lesions per total distance in each territory (diffusivity as described in Methods) performed better than lesion distance (line 8 versus line 7). Regarding their HRs, diffusivity in territories ranked as LM > RCA > LCX > LAD. A calcium-omics model with 39 features after elastic net regularization (line 9) was highly predictive of MACE with (HR = 3.62, *p* < 0.0001). When compared with other features (lines 1–8), the calcium-omics model had the best performance metrics in multiple categories. With the use of sampling to improve the event rate and elastic-net determination of 59 features, our calcium-omics model with sampling (line 10) yielded even better performance. Compared to the conventional standard (Agatston score, line 1) on held-out test data, this model improved C-index from 70.3% to 71.6% and the year-2 AUC from 68.8 to 74.8%. As these metrics are notoriously difficult to improve, we deem this increase substantive.

**Table 2:** Comparison of calcification risk models.

Cox PH model	Cox feature(s)	HR [± 95% CI; p-value]	Log-rank score	C-index (train) %	C-index (test) %	AUC (train) %	AUC (test) %
1. Agatston score	Ln (1 + Agatston Score)	1.39 [1.31,1.47; < 0.0001]***	157.4	71.3	70.3	71.8	68.8
2. Mass score	Ln (1 + Mass Score)	1.48[1.38, 1.59; < 0.0001]***	144.8	71.1	70.6	71.6	68.9
3. Arterial mass scores	Ln (1 + LM Mass)	1.13 [1.0,1.27; 0.034]*	138.6	71.2	**72.1**	71.8	69.3
	Ln (1 + LAD Mass)	1.27 [1.15,1.4; < 0.0001]***					
	Ln (1 + LCX Mass)	1.12 [1.0,1.25; 0.039]*					
	Ln (1 + RCA Mass)	1.1 [1.0,1.2; 0.062]					
4. Number of lesions	Ln (1 + Mass Score)	1.2 [1.03,1.41; 0.021]*	151.9	71.6	71	71.9	70.4
	Ln (1 + NumLesions)	1.48[1.34,1.92; 0.004]**					
5. Number of calcified arteries	Ln (1 + Mass Score)	1.39 [1.23,1.55; < 0.0001]***	145.2	71.3	70.8	71.6	69.7
	Is_ClacifedArteries > = 2	1.45 [0.91,2.31; 0.120]					
6. HUmax > = 1000	Ln (1 + Mass Score)	1.53 [1.42,1.67; < 0.0001]***	147.4	71.2	70.4	72.1	69
	is_HUmaxAbove1000	0.71 [0.51,0.99; 0.042]*					
7. Lesions’ distance (Top to last lesion)	Ln (1 + Mass Score)	1.38 [1.23, 1.54; < 0.0001]***	156.5	71.7	71.3	72.4	69.7
	LM distance	1 .001 [0.996,1.007; 0.617]					
	LAD distance	1.000 [0.997,1.003; 0.998]					
	LCX distance	1.003 [0.999,1.006; 0.102]					
	RCA distance	1.001 [0.999,1.003; 0.396]					
8. Territorial diffusivity	Ln (1 + Mass Score)	1.43 [1.32, 1.56; < 0.0001]***	154.2	71.8	71.6	72.5	70
	LM diffusivity	2.89 [1.05, 7.98; 0.041]*					
	LAD diffusivity	1.2 [0.26, 5.63; 0.815]					
	LCX diffusivity	1.52 [0.52, 4.43; 0.449]					
	RCA diffusivity	2.2 [0.4, 10.3; 0.310]					
9. Calcium-omics	Calcium-omics (39 features)	3.62 [2.92,4.48; < 0.0001]***	*185.9*	*74*	70.8	*74.6*	*71.4*
10. Calcium-omics (with sampling)	Calcium-omics (59 features)	2.81 [2.6,3.03; < 0.0001]***	**808**	**80.5**	*71.6*	**82.4**	**74.8**

To explain the role of particular features, especially high-risk features, we investigated multiple univariate and multivariable Cox models. Rows are models with different features or feature subsets. Columns are self-explanatory. We include results on both training and held-out testing data. The p-values are used to reject the null hypothesis that HR = 1 in the Cox model. See text for a detailed analysis of results.

P-values are star-coded based on the significance levels as follows: (< 0.0005 as ***, < 0.005 as **, < 0.05 as *).

Bold is the highest value, while italicized is the second highest value.

### Calcium-omics prediction comparison to traditional Agatston score

**Figure 4:**
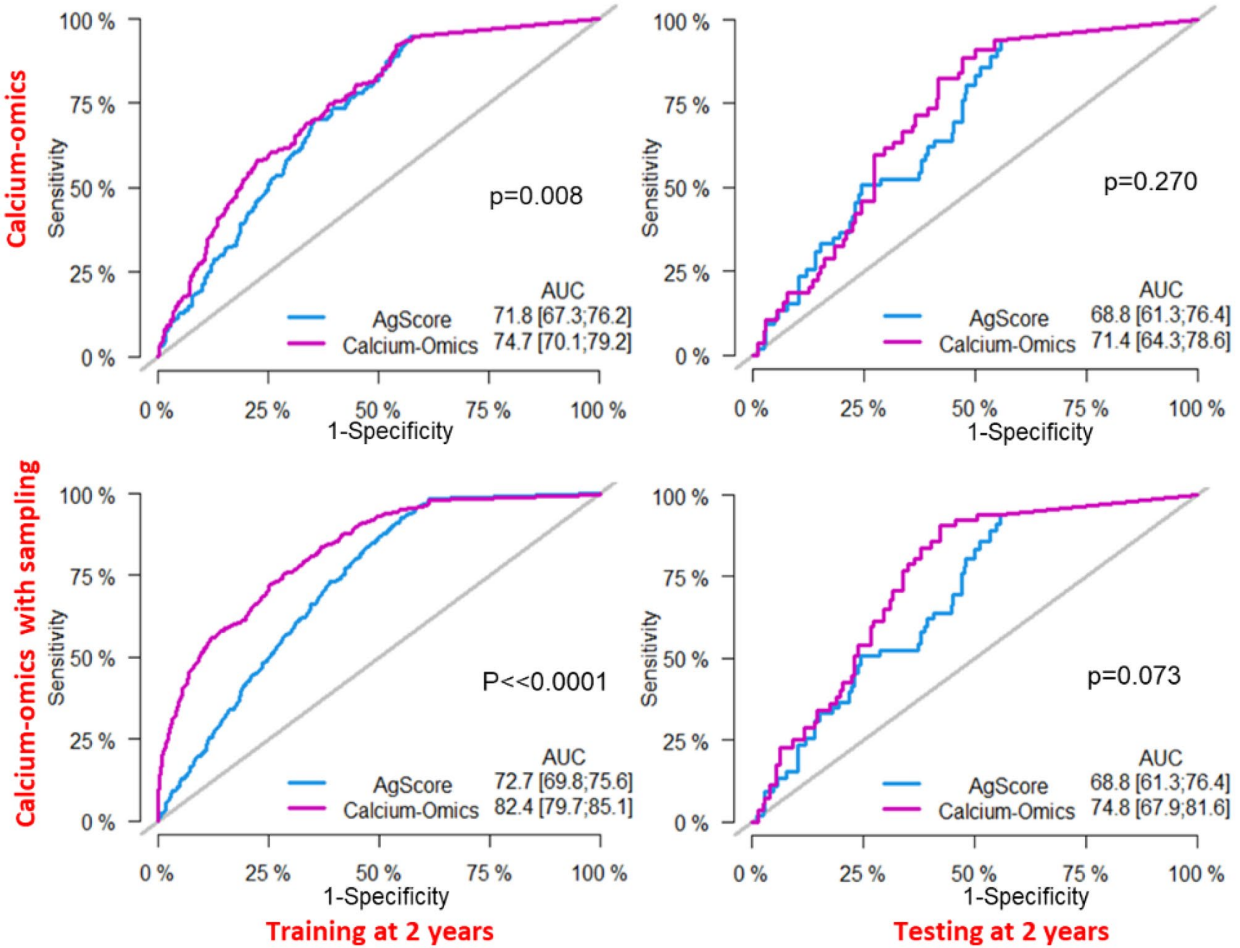
Performance of calcium-omics risk prediction with and without sampling (bottom and top, respectively). Along each row, calcium-omics ROCs are shown for training at 2 years, and testing at 2 years, respectively. Agatston score results are shown for comparison. Across the board, calcium-omics was superior to Agatston. Calcium-omics performance was improved with sampling and yielded a significant difference to Agatston (*p* <  < 0.0001) as compared to no sampling calcium-omics to Agatston (*p* = 0.008). For sampling, we used a modified-SMOTE (see text) with down sampling and up sampling on training data only. The held-out test set was not subjected to any data sampling strategy. The p-values correspond to the Wald test for the AUC significance of a model compared to the rival model.

In Fig. 4, we show year-2 ROCs for the calcium-omics model with and without sampling and compare them to the conventional Agatston score. Without sampling, calcium-omics gave (training/testing) AUCs of (74.7%/71.4%), while the Agatston model gave (71.8%/68.8%), respectively. Utilizing modified-SMOTE sampling, the calcium-omics model gave AUCs of (82.4%/74.8%), while sampling did not affect the Agatston score model. Similarly, at year-3, calcium-omics with sampling gave the best results. However, at year-3, there were fewer cases due to censoring and events, giving more uncertain results.

The Agatston model has a number of limitations. In contrast, the calcium-omics model is more discriminating due to its capacity to accommodate a broader range of calcium-omics features. While whole Agatston score had a non-linear relationship with MACE events in the log hazard ratio regression curve (Fig. S1), the calcium-omics model had a more linear curve. The calcium-omics model showed a wide range of risk levels for cases with similar Agatston scores in an interactive 2D surface regression plot (right plot in Fig. S1) implying good distinguishable values for cases having similar Agatston score.

**Figure 5:**
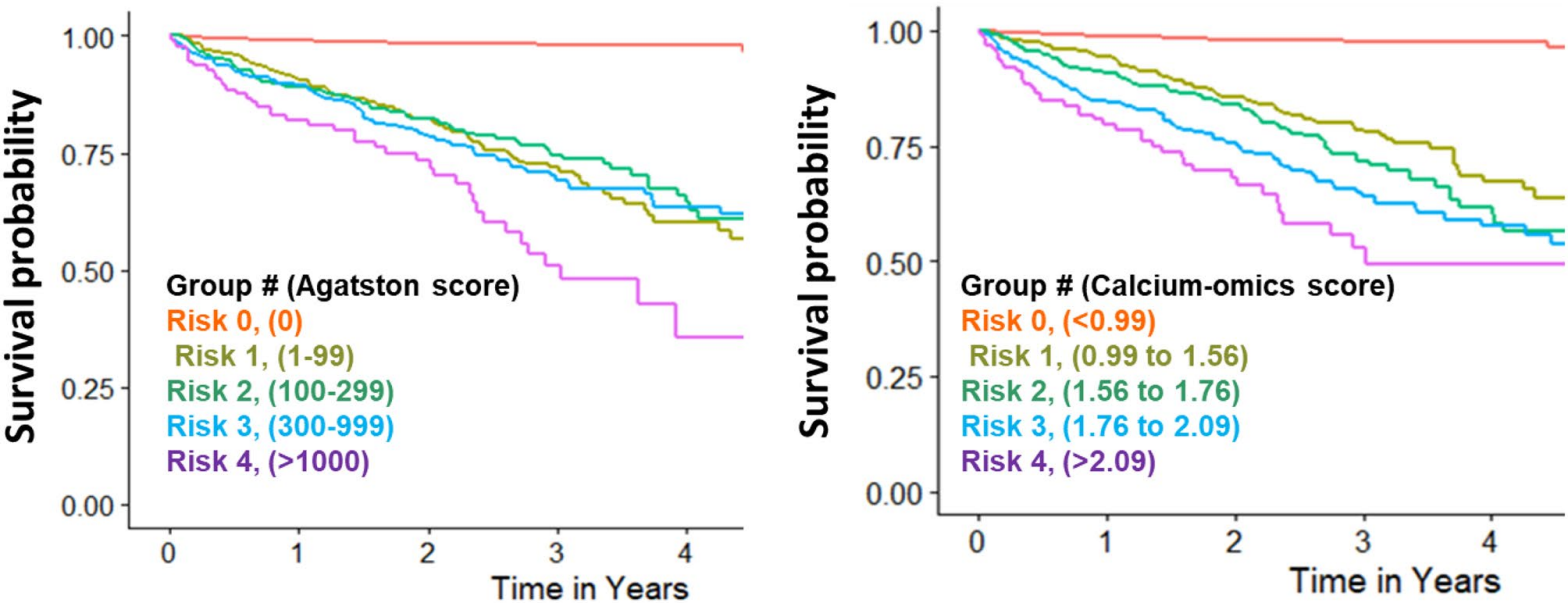
Kaplan Meier survival (MACE-free) curves with stratification provided by Cox modeling for all data. Plots represent the full MACE-enriched cohort using the standard Agatston score model (left) vs. calcium-omics model (right). The x-axis represents survival time, while the y-axis represents the survival probability of patients within a risk group. Agatston score was stratified into 5 groups according to the Lipid Association recommendation with Agatston score ranges (0, 1–99, 100–299, 300–999, and > 1000). Groups for the calcium-omics model were created with scores (< 0.99, 0.99–1.56, 1.56–1.76, 1.76–2.09, > 2.09) to give equivalent numbers of patients as for Agatston. The five risk severity groups are ordered (0–4), where 0 is the lowest-risk. Visually, the calcium-omics model much better stratified the five groups as compared to Agatston. In particular, risk groups 1, 2, and 3 are much more clearly separated for calcium-omics than Agatston. Due to the low number of held-out test samples, these plots are done with all data.

Consistent with the linear relationship between the calcium-omics model and MACE shown in Fig. S1, calcium-omics stratified risk groups better than did Agatston (Fig. 5). For the Agatston score (left), patients were stratified into the five risk groups recognized by the Lipid Association with Agatston score ranges (0, 1–99, 100–299, 300–999, and 1000 +)^14,15^. For calcium-omics, we set thresholds for the aggerated calcium-omics feature that gave the same proportions of patients in the risk groups as in the Agatston score plot. The calcium-omics model separated risk groups much better than does the Agatston model. Focusing on year two for Agatston, the three middle-risk curves are not very informative giving nearly the same MACE-free proportions. For calcium-omics at this time, there is informative, good separation. These results are consistent with the left and middle log hazard ratio regression curves in Fig. S1.

**Figure 6:**
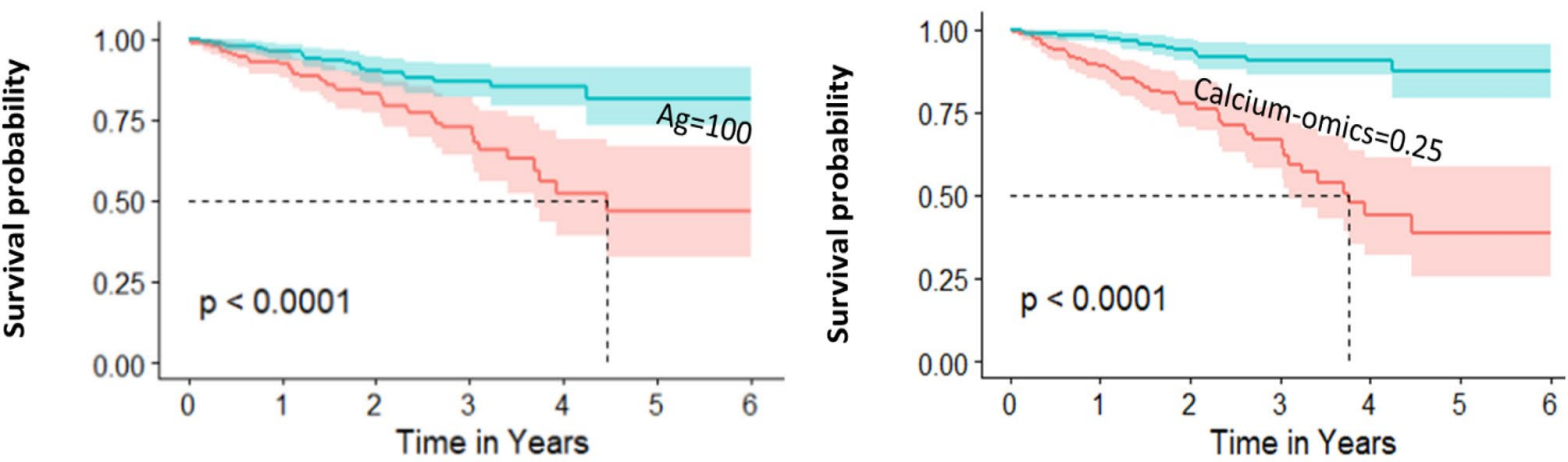
Kaplan Meier survival (MACE-free) curves with stratification provided by Cox modeling for held-out test set. Agatston score model stratified into low-risk (cyan) and high-risk (pink) risk groups based on below or above Agatston score of 100 (Left). In a similar ratio to the left model, the calcium-omics model stratified patients into low and high-risk (right) with a calcium-omics feature value = 0.25. The calcium-omics model showed better visual separable stratification by reclassifying some patients to fit into high or low-risk groups. Survival probability of 50% was reached at year 4.5 with Agatston model, while reached in 3.8 years in calcium-omics, showing advantageous to the latter model. At year four, we investigated the calcium-omics model categorical reclassification performance compared to Agatston score model. For the patients with MACE, the calcium-omics model showed a categorical net reclassification improvement of $NR{I}_{MACE}=0.132$. While with No-MACE patients, the new model showed $NR{I}_{NoMACE}=0.022$. The total NRI showed advantage to the new model with $NR{I}_{Total}=0.154 [95\% CI 0.006, 0.302; p=0.042].$ To conclude, the calcium-omics model identified 73.5% of MACE cases in the high-risk group, a 13.2% improvement as compared to Agatston, clearly showing high performance of the calcium-omics model for clinical decision-making.

Calcium-omics improved net reclassification in the held-out test set. Figure 6 shows Kaplan–Meier curves for the 20% held-out-test subset. As this smaller data set is insufficient to support 5-group stratification, we use a single cut-off of Agatston = 100, a value often considered in the literature. As before, the threshold for creating the calcium-omics curves gave the same starting patient proportions as for Agatston. Again, there is better separation provided by the calcium-omics model than the Agatston model. Considering results at year four, the calcium-omics model identified 73.5% of MACE cases in the high-risk group, a 13.2% improvement as compared to Agatston. The overall categorical net reclassification improvement was, $NRI=0.154 [95\% CI 0.006- 0.302;p=0.042]$, indicating improvement in the proposed model.

### Example patient highlighting calcium-omics advantage

Figure S2 highlights the limitation of the whole-heart Agatston score in two patients who have approximately equal Agatston scores (~ 204), but one has diffuse disease with 11 lesions in three territories, and the other has only two lesions in one territory. Whole heart Agatston would have predicted the same risk, but our calcium-omics approach predicts that at 3 years, the patient with only two lesions (right) will have a MACE-free survival probability 2.3 times better than the other patient with diffuse disease (left). The high-risk patient had a MACE later in the study period.

## Discussion

In this paper we provide an initial evaluation of an integrated radiomic approach that incorporates 80 different features spanning multiple elemental features of shape, texture, distribution and statistical parameters to predict MACE and compared this with the traditional Agatston score. The use of calcium-omics was far more discriminatory than Agatston, improving the AUC from 68.8 to 74.8 (*p* = 0.07). The relationship between an aggregated calcium-omics score and MACE was nearly linear with a graded effect, when compared to Agatston which displayed a non-linear relationship particularly at high levels of calcium score (Fig. S1). Importantly, calcium-omics resulted in a graded dose effects on MACE, as opposed to considerable overlap in risk across risk stratification quartiles (Fig. 5). While our findings are intuitive in the sense that incorporation of multiple features may be expected to enhance risk prediction, this is not always true given that many features may be correlated.

The success of calcium-omics relative to Agatston lies in its ability to better characterize coronary artery disease as compared to the Agatston score, which is simply a summation of calcium in the coronaries, albeit in a non-linear way. Calcium-omics captures characteristics from individual calcifications, including mass, volume, HU values, numbers of calcifications, numbers of territories, and spatial distribution. Univariate and multivariable Cox models in Table 2 offer explanation as to why calcium-omics does better, per the following observations. (1) Summing over the entire heart, mass score slightly improves prediction compared with Agatston, potentially due to its improved reproducibility^5,13^. (2) When we simply add the dense calcification (> 1000 HU) feature to mass, there is improvement as compared to mass or Agatston alone. Highly calcified “older” lesions are likely more stable explaining this finding^8^. (3) Adding mass scores from individual arteries improves performance. This suggests that having disease present in more than one artery is a risk factor. This is directly shown as a logical (≥ 2) in line 5 giving HR = 1.45 after accounting for total mass score. (4) Adding the number of calcifications to the whole heart mass score greatly improved risk prediction, again suggesting that the spread of disease is a risk factor. (5) Our diffusivity metric is a risk factor indicating that the spread of disease along arteries is a risk factor. Taking all features together in calcium-omics simply provides more information about the disease, enabling improved overall risk prediction.

Although the C-index is notoriously impervious to model improvements^11,12^, calcium-omics compared favorably to Agatston and gave a significant difference (*P* < 0.001). In addition to C-index, we consider it important to report other performance evaluations. As described in the Results, the calcium-omics model identified 73.5% of MACE cases in the high-risk group, a 13.2% improvement as compared to Agatston, suggesting that calcium-omics could be used to better identify candidates for intensive follow-up and therapies. The categorical net-reclassification index was NRI = 0.153.

There are important contributions of our work. Calcium-omics outperforms the current state-of-the-art, whole heart Agatston score. This development could contribute to more precise personalized therapies for cardiovascular patients. We purposefully used a MACE-enriched cohort in this preliminary study on AI analysis of calcium-omics features. The high event rate improved confidence intervals, allowing us to make more confident assessments of point estimates of HR values and of model comparisons, for this data set. In addition, this smaller cohort allowed us to carefully vet all data to ensure data quality. Overall performance might be different when larger numbers of cases with a low event rate are used in larger studies. We found that modified-SMOTE up sampling and down sampling reduces the problem with low event rate data. This is the first time that such strategies have been used with CT calcium score data. Often overlooked, down sampling and up sampling has been previously described for coronary heart disease cohort^16^.

Our study undoubtedly has limitations. Importantly, we had a limited observation period in our cohort (average 1.9 years within the 6-years study), which limits event rates. The data used in this study were from sites across the University Hospitals Health System, which is restricted to northeast Ohio. Other locales might have somewhat different results. Additionally, data used in this study were obtained using various scanners with similar acquisition parameters. We did not perform analyses to identify model performance by scanner type. Lead time bias is an inherent limitation in this study since MACE-free time is reported as duration from the time of CTCS exam until a patient either had MACE or was censored. This study primarily focuses on image-based classifications within coronary arteries, whereas comparisons to clinical-data-only models (e.g., MESA calculator and pooled cohort equation) will be part of future, more comprehensive studies on larger data sets. Our preliminary calcium-omics model used CT images and masks. Segmentation of coronary arterial calcifications is a labor-intensive and time-consuming task that is carried out by experts. It involves delineating each calcified lesion and assigning appropriate labels to the designated regions. Automation of this step is highly desirable.

In conclusion, we have obtained promising results using an AI analysis on detailed calcification features. Clearly, there will be advantage as compared to the standard whole-heart Agatston score. It is hoped that results will carry over to larger, confirming studies.

### Supplementary Information

Supplementary Information.

## References

[1] Beswick AD, Brindle P, Fahey T, Ebrahim S (2008). A Systematic Review of Risk Scoring Methods and Clinical Decision Aids Used in the Primary Prevention of Coronary Heart Disease (Supplement).

[2] Orringer CE (2021). The National Lipid Association scientific statement on coronary artery calcium scoring to guide preventive strategies for ASCVD risk reduction. J. Clin. Lipidol..

[3] Golub IS (2023). Major global coronary artery calcium guidelines. JACC Cardiovasc. Imaging.

[4] Osawa K, Nakanishi R, Budoff M (2016). Coronary artery calcification. Glob. Heart.

[5] Song Y (2023). Improved bias and reproducibility of coronary artery calcification features using deconvolution. J. Med. Imaging.

[6] Tota-Maharaj R (2015). Usefulness of regional distribution of coronary artery calcium to improve the prediction of all-cause mortality. Am. J. Cardiol..

[7] Brown ER (2008). Coronary calcium coverage score: Determination, correlates, and predictive accuracy in the multi-ethnic study of atherosclerosis1. Radiology.

[8] van Rosendael AR (2020). Association of high-density calcified 1K plaque with risk of acute coronary syndrome. JAMA Cardiol..

[9] Neves PO, Andrade J, Monção H (2017). Coronary artery calcium score: Current status. Radiol. Bras..

[10] R Core Team. R: A Language and Environment for Statistical Computing. R Foundation for Statistical Computing (2013).

[11] Hartman N, Kim S, He K, Kalbfleisch JD (2023). Pitfalls of the concordance index for survival outcomes. Stat. Med..

[12] Cook NR (2007). Use and misuse of the receiver operating characteristic curve in risk prediction. Circulation.

[13] Song, Y. *et al.* Improved reproducibility of CT calcium score using blind deconvolution. in *Medical Imaging 2021: Biomedical Applications in Molecular, Structural, and Functional Imaging* vol. 11600 116000V (International Society for Optics and Photonics, 2021).

[14] Agatston AS (1990). Quantification of coronary artery calcium using ultrafast computed tomography. J. Am. Coll. Cardiol..

[15] Ramanathan S (2019). Coronary artery calcium data and reporting system: Strengths and limitations. World J. Radiol..

[16] Datta G, Alexander LE, Hinterberg MA, Hagar Y (2019). Balanced event prediction through sampled survival analysis. Syst. Med..

