# Supplementary material for: Enhancing cardiovascular risk prediction through AI-enabled calcium-omics
Source: Sci Rep. 2024 May 15;14:11134. doi: 10.1038/s41598-024-60584-8 (PMC11096314; doi:10.1038/s41598-024-60584-8)
Supplement: Supplementary file 1 — Supplementary Information. [file 41598_2024_60584_MOESM1_ESM.pdf]

S.1 Detailed feature engineering

The three main traditional whole-heart scores (Agatston, mass, and volume) are given below.

1. Agatston score. Agatston score uses a weighting factor depending upon the maximum HU value for each lesion in a 2D CT image. The total Agatston score was calculated by summing the product of the density weighting factor (DWF) and the 2D area of each calcified lesion. (Some larger 3D lesions will have multiple 2D entries.) The whole heart Agatston score is obtained as given below.

$$Agatston\ score = \frac{3}{ST}\sum_{i=1}^{N} DWF(MaxHU_i) \times\ Area_i \tag{1}$$

Here, N is the number of 2D lesions, $MaxHU_i$ is the maximum HU value within the i-th lesion. $Area_i$ is the i-th lesion 2D area in mm^2. As the Agatston score was originally calculated with a $3\ mm$ slice thickness, we adjust values with the ratio, $3/ST$, where ST is the new slice thickness. Values for $DWF(\ )$ are given below [19].

$$DWF(x) = \begin{cases} 1, & 130 \le x < 200 \\ 2, & 200 \le x < 300 \\ 3, & 300 \le x < 400 \\ 4, & 400 \le x \end{cases} \tag{2}$$

2. Mass score. The absolute mass of coronary calcium score is aggregated per lesion with the aid of phantom calibration and evaluated as follows:

$$Mass\ score = \sum_{i=1}^{N}\sum_{v=1}^{M} k \times HU_v \tag{3}$$

where k is a calibration factor converting HU to mg. N is the number of lesions. M is the number of voxels within lesion i, and HU_v represents the HU value of the selected voxel v. For a Philips scanner at 120 kVp, k = 0.71 $(mgHA/cm^3)/HU$.

3. Volume score: Volumes were obtained by simply summing labeled voxels $v = [1,..,M]$, and multiplying the volume per voxel, V.

$$Volume\ score = VM, \tag{4}$$

In addition to whole heart aggregated features (such as Agatston score, Volume score, and mass score), we collected lesion, lesion-to-lesion, and arterial-wise features. We calculated per artery score features, including Agatston score, mass score, and volume score. We engineered more calcium-driven features such as lesion aggregated areas, HU statistical features (min, max, average, median, and standard deviation), distance from the first slice to last calcification, and distance from first to last lesion along descending arterial lesions. We also collected lesion-based statistical histogram bins of the first moment, second moment, mean moment, skewness moment, kurtosis moment, and average HU. Some of these features are briefly explained as follows, where 2D features are slice-based, and 3D features are volume-based:

1-***Numerical*** features include:

- **Area2D** (total heart summation of lesions' areas across all slices)
- **NumLesion3D** (total heart number of 3D lesions)
- **numLesionPerArtery3D_<<*name*>>1** (num of 3D lesion in specified artery)
- **AgatstonScore2D** (heart total Agatston score calculated in slice-based lesions, original Dr. Agatston approach)
- **AgatstonScore3D** (heart total Agatston score calculated in 3D volume-based lesions)
- **AgatstonScorePerArtery2D_<<*name*>>1** (<<*name*>> artery Agatston score calculated in slice-based lesions)
- **MassScorePerArtery_<<*name*>>1** (<<*name*>> artery mass score)
- **VolumeScorePerArtery_<<*name*>>1** (<<*name*>> artery volume score)
- **massHist<<number>>** (histogram bin <<number>> out of 5 bins of lesions-based mass score)
- **avrHist<<number>>** (histogram bin <<number>> out of 5 bins of mean HU values)

- **DistTop2LastLesionPerArtery_<<name>>1** (Euclidean distance summation in mm, starting from center of top CT slice along centroid of each consecutive lesion till last lesion within <<name>> artery)
- **DistFirst2LastLesionPerArtery_<<name>>1** (Euclidean distance summation in mm, starting from centroid of first lesion, along centroid of each consecutive lesion till last lesion within <<name>> artery)
- **ICfirstMomentH<<number>>** (max values of first momentum among individual calcifications, order <<number>>) (<<number>> up to 3 values)
- **ICsecondMomentH<<number>>** (max values of second momentum among individual calcifications, order <<number>>) (<<number>> up to 3 values)
- **ICmeanMomentH<<number>>** (max values of mean momentum among individual calcifications, order <<number>>) (<<number>> up to 3 values)
- **ICskewnessMomentH<<number>>** (max values of skewness momentum among individual calcifications, order <<number>>) (<<number>> up to 3 values)
- **ICkurtosisMomentH<<number>>** (max values of kurtosis momentum among individual calcifications, order <<number>>) (<<number>> up to 3 values)
- **HUperArtery2D_stat<<name>><<number>>** (<<number>> [1-4] represents [min, max, mean, std] statistical values of Hounsfield Units of each calcified voxel within artery<<name>>)
- **<<name>>_diffus** (factor indicates diffusivity of lesions within <<name>> artery, calculated as the ratio of number of lesions to Euclidean distance along lesions with artery from first to last lesion. We considered the non-calcified artery to have zero diffusivity while the single lesion artery to have diffusivity=one)

2-***Categorical and conditional*** (Boolean) features include:

- **isAgZero** (is Agatston score equal zero?)
- **isLesion3DBelow5** (is number of lesions less than 5?)
- **AgGroupX1-X3**, Agatston score groups of (0,1-99, 100-399, 400+) represented in three (X1, X2, X3) Boolean digits to be used in Cox.
- **isArt2plus** (are there two or more calcified arteries?)
- **isArt3plus** (are there three or four calcified arteries?)
- **numArtCalc** (number of calcified arteries 0-4)
- **HU1000** (Does the patient have any calcified lesions with HU value above 1000?)

These image-based engineered features are listed in Table S1. We exclude features that are clinical or highly correlated. Among the remaining 61 features, an elastic net with 10-fold cross-validation selected 40 features, as indicated, with their corresponding Cox model coefficient values. The elastic-net Cox proportional hazard model was deemed the Calcium-omics model.

S.2 Time-to-event modeling with Cox proportional hazard model and elastic-net regularization

For a clinical study at a fixed time with persons entering at various times, censoring of the observation time is an issue requiring time-to-event modeling rather than binary classification. A time-to-event model estimates the probability that the event (MACE in our study) may have occurred during a follow-up period. Whether the patient had an event or being censored, data can be modeled by a distribution function [20] of observed time T, at a patient survival time t, called the cumulative incidence function:

$$F(t) = P(T < t) = \int_t^\infty f(u)du, \tag{5}$$

where$f(t)$ is the probability density function. $P(T < t\ )$ is the probability function that survival time is less than t. The survival function $S(t)$, is $1 - F(t)$, which is the probability that the time T is greater or equal to t.

$$S(t) = P(T \geq t) = 1 - F(t). \tag{6}$$

The hazard function is represented as the risk of hazard of an event occurring at time t and is defined as:

$$h(t) = \frac{f(t)}{S(t)}\,. \quad (7)$$

The Cox proportional-hazard regression model [21] is widely used in survival modeling. The Cox model provides a semi-parametric hazard rate of each covariate in the model, as follows:

$$h(t|\mathrm{A}) = h_0(t)\exp(\beta_1 a_1 + \beta_2 a_2 + \cdots + \beta_n a_n), \quad (8)$$

where $h_0(t)$ is the baseline hazard, $A = [a_1, a_2, \dots a_n]$ is the covariate feature vector of n features, β_i is the i_{th} covariate coefficient. The Cox model is optimized using maximum-likelihood. We used Cox regression for univariate and small multivariable models to study feature effects and identify high risk features. There are practical considerations. So as not to over-emphasize large covariate values, we compress dynamic range by taking a logarithm of some covariates (e.g., log (Agatston Score)). As Cox modeling is sensitive to correlated features, results may not reflect the actual effect of one feature over another. Too many features can result in over-fitting. We used elastic-net regularization with cross-validation [22] to select the best features.

To overcome the effect of low event rates [18], we applied down sampling followed by up sampling techniques on the majority and minority class, respectively. We used a modified Synthetic Minority Oversampling Technique (modified-SMOTE) approach. For major class down sampling, we used few continuous features (e.g., Agatston score, mass score, and volume score) to determine eligibly samples to be removed using k-nearest neighbors (KNN) (k=5) in feature space. For up-sampling, we created synthetic instances “nearby” actual samples in “covariate space.” Briefly, we used similar features (as in down sampling), synthetic instance was inserted within KNN (k=5). For the new sample, continuous feature value was calculated as the median of the corresponding k-neighbors feature value, while non-continuous (logical and categorical) feature values were copied from the nearest single neighbor. The new instance time-to-event was randomly set. Down sampling was done until MACE events increased from 13.8% in the original data to 16.4% by removing 20% of the No-MACE cases. Followed by up sampling, new cases were inserted until the MACE events increased to 30%. We never applied up or down-sampling on held-out test data.

S.3 Comparison between Agatston and Calcium-omics Cox models

Whole Agatston score had a non-linear relationship with MACE events in the log hazard ratio regression curve (Fig. S1), the calcium-omics model had a more linear curve. These curves were plotted using the Cox model of penalized spline of a feature and calculated the log of hazard ratio of each patient to show the distribution along the regression curve. The calcium-omics model showed a wide range of risk levels for cases with similar Agatston scores in an interactive 2D surface regression plot (right plot in Fig. S1) implying good distinguishable values for cases having similar Agatston score. The contours in this plot delineate areas that correspond to equivalent levels of disease severity. Interestingly, the plot shows the capability of calcium-omics to cover a wide variation of values for narrow Agatston score values.

Figure S2 shows the advantage of calcium-omics over Agatston score model for two patients with approximately equal Agatston scores (~204), but one has diffuse disease with 11 lesions in three territories (left), and the other has only two lesions in one territory (right).

Selected 61 engineered features (elastic-net selected only 39, shown with coefficients)

Feature	***coef***	***Feature***	***coef***	***Feature***	***coef***	***Feature***	***coef***
MassScore*	-0.6812	VolumeScore*	-0.0774	Area2D*	1.21	NumLesion3D*	-0.9914
isAgZero	-	isLesion3DBelow5	-	AgGroupX1	-	AgGroupX2	-
AgGroupX3	-	numLesionPerArtery3D_LM1*	0.07381	numLesionPerArtery3D_LAD1*	-	numLesionPerArtery3D_LCX1*	-
numLesionPerArtery3D_RCA1*	0.1398	isArt2plus	-	isArt3plus	-	numArtCalc	-0.1718
AgastonScorePerArtery2D_LM1*	0.1083	AgastonScorePerArtery2D_LAD1*	-0.2756	AgastonScorePerArtery2D_LCX1*	-0.1445	AgastonScorePerArtery2D_RCA1*	-0.07467
MassScorePerArtery_LM1*	-	MassScorePerArtery_LAD1*	0.4396	MassScorePerArtery_LCX1*	0.3636	MassScorePerArtery_RCA1*	0.1652
VolumeScorePerArtery_LM1	-	VolumeScorePerArtery_LAD1	0.00048	VolumeScorePerArtery_LCX1	-0.00101	VolumeScorePerArtery_RCA1	-
massHist1	2.332	massHist2	2.152	massHist3	0.7786	massHist4	2.521
massHist5	1.366	avrHist1	0.7336	avrHist2	0.1187	avrHist3	-0.4072
avrHist4	-	avrHist5	0.8539	DistTop2LastLesionPerArtery_LM1	-	DistTop2LastLesionPerArtery_LAD1	-
DistTop2LastLesionPerArtery_LCX1	0.00679	DistTop2LastLesionPerArtery_RCA1	0.00358	DistFirst2LastLesionPerArtery_LM1	-	DistFirst2LastLesionPerArtery_LAD1	-
DistFirst2LastLesionPerArtery_LCX1	0.00062	DistFirst2LastLesionPerArtery_RCA1	-	ICfirstMomentH1	-0.01407	ICfirstMomentH2	-0.00435
ICfirstMomentH3	-0.0073	ICsecondMomentH1	4.9E-05	ICsecondMomentH2	-	ICsecondMomentH3	-
ICmeanMomentH1	0.04454	ICmeanMomentH2	-	ICmeanMomentH3	-	ICskewnesstMomentH1	-0.01654
ICskewnesstMomentH2	0.00288	ICskewnesstMomentH3	-0.0002	ICkurtosisMomentH1	0.3444	ICkurtosisMomentH2	0.02595
ICkurtosisMomentH3	-0.3646						

Excluded features before using elastic net

Feature	***Feature***	***Feature***	***Feature***
AgatstonScore2D*	AgatstonScore3D*	HUperArtery2D_stat_LM2	HUperArtery2D_stat_LM3
HUperArtery2D_stat_LM4	HUperArtery2D_stat_LAD2	HUperArtery2D_stat_LAD3	HUperArtery2D_stat_LAD4
HUperArtery2D_stat_LCX2	HUperArtery2D_stat_LCX3	HUperArtery2D_stat_LCX4	HUperArtery2D_stat_RCA2
HUperArtery2D_stat_RCA3	HUperArtery2D_stat_RCA4	LM_diffus	LAD_diffus
LCX_diffus	RCA_diffus	HU1000	

* This feature was represented in logarithmic function as log(x+1)

Table S1. List of all selected and excluded (before using elastic-net) image-based calcification-driven features. 19 features were excluded prior to the proposed model design due to their high correlation with other features. We used them in designing univariate and multivariable Cox models to investigate and compare with other models (such as Agatston score, HU1000, and LAD_diffus). Among the 61 listed features, an elastic net with 10-fold cross-validation selected 39 features, as indicated, with their corresponding Cox model coefficient values. The elastic net Cox proportional hazard model was deemed the calcium-omics model. These features were used to design the calcium-omics Cox model using training data without sampling.

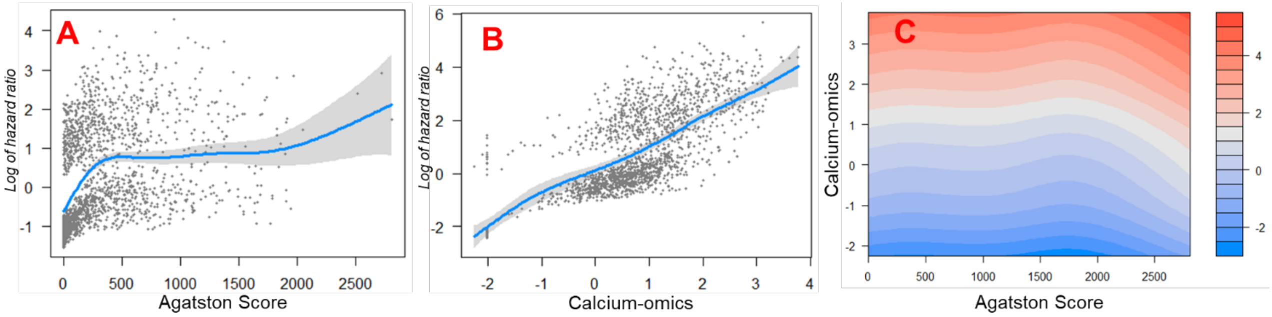

Figure S1. Log hazard ratio, i.e., ln[h(t)/h_0(t)], regression plots for Cox models as a function of Agatston (A) and calcium-omics scores (B). Visualizations are available using the *visreg( ),* and *visreg2D( )* functions in *visreg* R Library. Briefly, we used penalized spline (*pspline*) function to create the blue log hazard ratio curves. Each data point represents a patient's deviance residual, and the shaded-gray areas show the 95% CI. As compared to the Agatston model, the calcium-omics model shows a desirable, linear distribution along the data points. In the case of the Agatston model (A), a wide range of Agatston score (300-2000) gives very similar results. Similar observations are shown in (C), where the log hazard ratio is displayed in gradient-colored contours, from low (blue) to severe (red). Calcium-omics is platted as a function of Agatston. At a given value of Agatston, there is considerable variation of the log hazard calculated from calcium-omics. For example, with an Agatston score of 500, several levels of severity are covered by the calcium-omics model. This suggests added value.

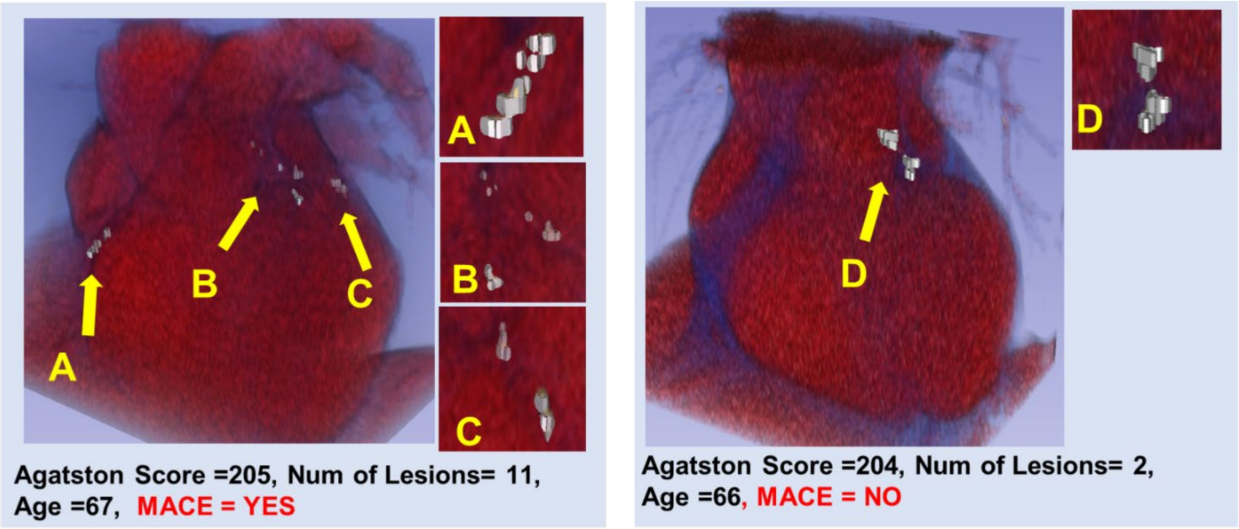

Figure S2. Whole-heart Agatston does not reflect the spread of disease and risk for these two patients, both with a whole-heart Agatston score of ~204. The left heart has 11 calcifications spread throughout the heart (i.e., LAD:6, LCX:3, and RCA:2), with Agatston scores of (29.6, 84.4, and 90.8), respectively. The right heart has two “nearby” large calcifications (LM:1, LAD:1) with Agatston scores of (108.2 and 95.6), respectively. Both patients are from the held-out test set, with the same Age (~67). Despite an equal whole-heart Agatston score, the calcium-omics model described later predicted a 3-year risk for the left heart 2.3 times that of the right heart. The patient on the left patient had a MACE event, while the right did not have MACE.
